# Community perceptions on the secondary health benefits established by malaria vaccine trials (RTS,S phase 2 and phase 3) at the Korogwe site in North Eastern Tanzania

**DOI:** 10.1186/1475-2875-12-157

**Published:** 2013-05-08

**Authors:** Edwin A Liheluka, John P Lusingu, Rachel N Manongi

**Affiliations:** 1 National Institute for Medical Research, Tanga Centre, Korogwe site, Tanga, Tanzania; 2 Kilimanjaro Christian Medical University Collage (KCMUC), Kilimanjaro, Tanzania

**Keywords:** RTS,S malaria vaccine, Secondary health benefits, Community perceptions

## Abstract

**Background:**

Studies conducted thus far have demonstrated that the malaria vaccine (RTS,S) has a promising safety profile. Within the context of planning for future vaccine trials and for the purpose of building on previous research that has been undertaken in sub-Saharan Africa with regard to community perceptions about clinical studies, this research aimed to explore the community perceptions on the secondary health benefits established by the malaria vaccine trials (RTS,S Phase 2 and Phase 3) at the Korogwe site in Tanzania.

**Methods:**

An exploratory qualitative study design was used. Participants were recruited from the Korogwe site. Sampling techniques were purposive and random. A total of five focus group discussions and six in-depth interviews were conducted. Interview guides with open-ended questions were employed to collect data. Male and female parents whose infants participated and those whose infants did not participate in the trials, health workers and community leaders were interviewed. Thematic analysis framework was used to analyse the data.

**Results:**

The activities of a malaria vaccine project appeared to be well known to the community. Respondents had largely positive views towards the secondary health benefits which have been established by malaria vaccine trials. The project has led to a massive investment in health care infrastructure and an improvement in health care services across the study areas. The project was perceived by the community to have established major secondary health benefits. Misconceptions amongst respondents, especially with regard to blood samples, were also observed in this study.

**Conclusion:**

Despite some misconceptions with regard to the conduct of malaria vaccine trials, especially on blood sampling, generally this study observed that most participants were positive about the secondary health benefits brought about by the malaria vaccine trials in Korogwe.

## Background

The world today is proud of the success and achievements that have been made in clinical development through research conducted around the world. Many diseases are being cured and many are prevented due to the presence of vaccines and other interventions. In low-income countries more vaccines are now available and more lives are being saved, with a significantly decreased number of children dying each year due to the increased immunization coverage [1].

Clinical research plays a vital role in complementing the overburdened national health delivery system, especially in rural, resource-poor settings [2]. A malaria vaccine is among the promising new vaccines that are in the final stages of research. The development of this vaccine will save millions of lives, mostly in tropical settings.

Malaria persists as a major public health problem, and new tools for controlling the disease are needed to facilitate the current renewed commitment for its control or elimination [3]. Optimal control of malaria disease may be met through a combination of methods such as vector control [4], chemotherapy [4] and/or vaccines [4]. The challenge facing current control efforts, however, is the development of resistance to anti-malarial drugs and insecticides [4].

The primary health benefits of the malaria vaccine trial (MVT) refer to the major profits or gains that an individual acquires after having been vaccinated with the malaria vaccine, i.e. the efficacy and effectiveness of the malaria vaccine [5].

The secondary health benefits of the MVT refer to the additional benefits that an individual or community receives due to the presence of a malaria vaccine project in a certain geographical area. Examples of secondary health benefits may include: community health education by research staff; community access to quality and free medical care; provision of ambulance services to the community and provision of transport [5].

Several studies that have been conducted worldwide on community perception regarding clinical trials reported different findings. A study done in Papua New Guinea [6] on “Community response to intermittent preventive treatment of malaria in infants (IPTi)” reported that parents of participants in that study commented that their participation had brought them broader benefits as they travelled less often to the clinic since their children had been treated and protected by the trial medicine, and gave them more time for other activities such as farming. Indeed, mothers commonly enrolled more than one of their children in the study because of the benefits obtained from the study. For example, trial staff were considered to be diligent and caring compared to government health staff who were rude, slow to deal with mothers and did not give proper care. One mother conceded that government health workers were rude because they have a heavy workload. This study reported both the primary and secondary benefits of the malaria drug trial [6]. In addition the IPTi malaria trial was able to distribute new bed nets to all participants in the trial. The provision of a bed net was perceived by the community as a secondary health benefit established by the IPTi trial [6].

A study done in Malawi [2] on “Why mothers choose to enrol their children in malaria clinical studies and involvement of relatives in decision making”, reported that the majority of respondents joined the intermittent prevention therapy post-discharge (IPTpd) malaria research due to the secondary health benefits, such as access to quality medical care, rather than the investigative aspects of the research. They also decided to enrol their children in order to benefit from the material and monetary incentives which included soap, peanut butter, orange drink, transport money, napkins, mosquito nets, basins and iron tablets that were being given to participants by the project.

Another study in Kilifi on the Kenyan coast [7] on “Community understanding and perceptions of a malaria vaccine trial (MVT)” reported that the trial was generally described as some form of assistance, as a project aimed at ensuring good health in children through prevention, treatment and check-ups. This was also true for those who apparently understood the research aims of the trial. Linked to this finding, the main reasons for joining and staying in the study were reported by most to be the individual benefits associated with the study, such as free treatment, transport to and from Kilifi District Hospital in case of any illness and access to the Principal Investigator (PI), who is medically qualified.

Lusingu, the Principal Investigator of the current phase 3 MVT in Korogwe, reported during an interview with “one blog” that MVTs have led to a massive investment in health infrastructure in the communities where vaccine trials have been implemented. For example, a fully equipped modern laboratory that specializes in parasitology, haematology, biochemistry and microbiology has been built in the study area (Korogwe) and is now fully functional. Vaccine ‘cold chain,’ X-ray services, paediatric care and referral systems have all been enhanced in the study area. Important health services have also been provided to both participants and non-participants in the trial and community members’ compliance with treatment and attitudes toward health-seeking behaviour have improved [8]. This personal interview in Korogwe reported the secondary health benefits of the MVT but focused only on the provider perceptions and not community perceptions. This MVT has been able to employ more than one hundred staff, who have different levels of training ranging from secondary education to masters level.

Understanding community perceptions on the secondary health benefits of this MVT is crucial and the findings of this study will add to the body of knowledge already available in the literature by highlighting the secondary health benefits of the MVT in the community, which may prompt active community involvement in future clinical trials.

Results from this study will provide additional evidence to relevant stakeholders, such as malaria vaccine manufactures, sponsors and policy makers to guide MVTs, particularly in Tanzania and throughout Africa. This paper reports the community perceptions on the secondary health benefits established by the MVT in the Korogwe site, Tanzania.

## Methods

### Study design and area

A qualitative study design was chosen to explore the community perceptions on the secondary health benefits established by the MVTs in the Korogwe site, Tanzania. This study was conducted between April and May 2012 and was conducted in the communities involved in the phase 2 and 3 RTS,S MVTs. Korogwe site is located in north-eastern Tanzania. This research centre started to conduct research in 2000 and since then a wide range of biomedical research has been done. The population of Korogwe district according to the 2002 Tanzania National Census Survey was 261,004, with a growth rate of 1.4% per annum. The district has 47 Government dispensaries, fivr health centres and two hospitals. The RTS,S phase 2 and phase 3 MVTs have built nine vaccination clinics across the study area in Korogwe District, which covers 34 study villages. Korogwe District can be topographically stratified into lowland and highland zones, with altitudes ranging from 300–1,200 metres above sea level. Malaria transmission in these areas decreases with increasing altitude and varies within a short distance. In both zones, malaria transmission is highest during and following the long rainy season, which usually extends from March through July.

The first MVT in Korogwe was RTS,S phase 2 which was conducted between 2006 and 2008. This phase 2 study was conducted in 17 villages which were served by three satellite vaccination clinics and the paediatric ward at Korogwe District Hospital (KDH). The RTS,S phase 3 MVT started in 2010; this study operated in 34 villages in Korogwe and Handeni Districts, including the previous 17 villages involved in phase 2, and they were also served by nine vaccination clinics and KDH.

### Study population

The study population included parents or caretakers of children below five years of age registered in the MVT database and key informants in the study area. Participants who were selected to take part in this study were caretakers at the Korogwe site who had been there during the implementation of the phase 2 and phase 3 MVTs. Respondents were 18 years and above and were able to give consent. Participants were excluded if they were away from the study area during the interview period and if they were seriously sick during the interview.

### Sampling techniques and Data collection methods

A random selection technique was used to select villages and study participants for focus group discussions (FGDs) from an already existing study database. Purposive sampling technique was used to select participants for in-depth interviews (IDIs). Of 34 villages that participated in the MVT, only five were randomly selected using the Excel function known as “randomize between” to sample participants for FGDs. Although this is a qualitative study, random selection technique was used to select participants because of the availability of a huge census database with all the information on all 34 study villages. From the list of potential participants from five randomly selected villages, those who did not participate in the MVT were entered differently in Excel. Each list was again sorted to differentiate between female and male participants. Thereafter, the Excel function “randomize between” was used to produce 12 female and male participants from the MVT respectively in the two different lists. The same procedure was done for those who did not participate in the MVT and produced 12 participants. The purposive technique was used to select six key informants to participate in IDIs. The criteria for selection were people from the Korogwe site who were knowledgeable about the study topic. The selected participants included two health workers who had observed the activities of MVTs since 2006, so they had sufficient knowledge about the topic, and the four other participants were village leaders who were involved during the sensitization meetings prior to the implementation of the study.

FGDs and IDIs with key informants were used to collect data. Before the interview, demographic information on participants was collected and subject numbers were given to participants chronologically.

### Conducting focus group discussions and In-depth interview

FGDs were used to obtain information about community members’ perceptions on the secondary health benefits of MVTs. After randomization of participants a day before the interview, the PI travelled to the respective villages and, with the help of the community leader, identified the participants and invited them to attend the meeting. The venues for the FGDs were organized with assistance from local village leaders in each village. Prior to the discussion, the importance of participating in the study was explained and participants were given informed consent forms (ICF) to sign. Impartial witnesses were used for those deemed illiterate. A copy of their signed ICF was offered to each participant.

Each FGD comprised eight to eleven participants. All FGDs were conducted in a ‘round table’ manner in the participants’ respective villages. The PI facilitated the FGDs while the research assistant experienced in qualitative note taking took notes. The interview guide was used with flexibility during the FGDs.

The PI was keen to probe for more information in order to bring to life the discussions amongst participants. This process continued until participants were satisfied they had no further points to add. The PI stopped the interview and thanked the participants for their participation. After each interview, the facilitator and note taker expanded the notes before moving to another group in order to observe what information was missing or contradictory, so that this could be incorporated in the next FGDs. Two FGDs were conducted per day in each village.

One IDI with a key informant took 33 to 51 minutes. Interview guides with open-ended questions were prepared and the PI asked the questions as per the guide. IDIs were conducted in a private setting convenient to the participants. FGDs were conducted before IDIs in order to get an overview of the subject matter before interviewing key informants. Information gathered during the FGDs was incorporated into the IDI guide and was explored further during the IDIs.

### Data process and analysis

Data was analysed manually using thematic analysis framework. According to Braun and Clarke [9], thematic analysis framework has six main steps, namely familiarization of the data, generating initial codes, searching for themes, reviewing themes, defining and naming themes and producing the report. For the sake of capitalizing on validity and reliability, all collected information in the field was given a code for confidentiality. The triangulation method was used to collect data. The PI, with the help of the note taker, read the notes and expanded them before moving to another group, in order to examine what information was missing so that it could be incorporated in to the next FGD. The field notes were read regularly during and after discussions in order to confirm that all points discussed had been noted accurately. The narratives were read and coded by an experienced independent researcher from Kilimanjaro Christian Medical University College (KCMUC), themes were then compared, discussed and agreed upon together with the PI.

## Ethical approval

Ethical clearance was granted by Kilimanjaro Christian Medical University College Ethics Committee with clearance number 456. A letter from the Regional Administrative Secretary was presented to the administration in the study district. All participants in the IDIs and FGDs received information about the research and about the voluntary nature of the research, both verbally and in writing, before signing a consent form to comply with the regulations of the Tanzania National Health Research Forum.

### Results presentation

Descriptive indicators [10] were used to present the findings of the study. The following explanations detail how the results are presented in percentages: all (100% of respondents); nearly all (80-90% of respondents); a majority (more than 50% of respondents); fewer than half (around 25-45% of respondents); a minority (10-25% of respondents) and a few (less than 10% of respondents).

“A” represents participants in the FGDs and “B” represents participants in the IDIs. Subject numbers were given to participants chronologically.

## Results

### Demographic characteristics of study participants

Of a total of 55 participants, 49 participated in the FGDs and six participated in IDIs; all were male. Most participants resided in rural areas. Among respondents, small-scale farming and small businesses were common occupations. Those with formal employment included MDs and VEOs. Respondents’ ages ranged from 21 to 71 years.

Participants had an overall positive view towards the secondary health benefits established by MVTs. The main themes that emerged from this study were: i) improvement of health care services; ii) transport; iii) infrastructure and iv) human resources. Misconceptions were also reported by a minority of participants

### Improvement of health care services

All participants agreed that the phase 2 and phase 3 RTS,S MVTs improved the quality of health care to the study participants, as well as the community as a whole, by providing reliable, full-time and quick medical care. The participants claimed that the project had improved health care services at the hospital level where a modern laboratory and radiology unit had been built in the compound of Korogwe District Hospital. The presence of these facilities offered better health care to study participants, as well as to the whole community. One 36-year-old male health worker explained that:

“*We have a big laboratory which has been built by the malaria project through donors’ fund, for this laboratory to be in the District Hospital compound, it has given us (the District Hospital) the opportunity to utilize the structure, human resources as well as the services and because of the poor quality of most of the government laboratories, the presence of this laboratory which is also monitored and overseen by the laboratory professionals and experts from abroad, it has helped us so much to have the quality laboratory services in the District”* (Participant number B55)

Participants who did not get an opportunity to participate in the MVT expressed their willingness to participate in the project because of the quality health care services offered by the project. A 42-year-old female who did not participate in the trials explained that:

*“Those who have been enrolled in the malaria vaccine project are really praising the project; I want also to join the project because of the good health services which are being offered by the project such as free medical care*…” (Participant number A3)

Some participants who participated in the FGDs but who did not participate in the trials were not aware of the health care services being offered by the project.

The majority of respondents, including those who participated in the MVT and those who did not, stated that the project has been able to reduce the number of deaths for children under five in the study area due to the effective and fast treatment offered to trial participants as well as non-participants. A 36-year-old male health worker explained:

*“The death of children has been massively reduced in the paediatric ward”* (Participant number B5)

Some respondents who participated in the FGDs but who did not participate in the MVTs, were quiet about this subtheme, likely because they were not knowledgeable about the topic.

The majority of the participants, especially women in the FGDs, reported that the malaria vaccine project provided treated mosquito nets to all participants. However, there were concerns about the number of nets provided by the project, which was perceived to be limited. A minority of the participants did not comment on this subtheme. One 59-year-old female commented that:

*“…If possible we request that each participant in the malaria vaccine project should get more than one bed net in order to prevent other children in the family…”* (Participant number A09)

In addition to distribution of bed nets, most participants mentioned close follow up and monitoring of participants by the project health workers as an important component of the project. A 53-year-old male participant stated:

*“…Participants in the malaria vaccine project are being followed by the project field workers to monitor their health status…”* (Participant number A19)

Nearly all respondents expressed that the malaria vaccine project provided very good care to trial participants. Project staff use polite language and provide good care to participants and non-participants. However there are some malaria vaccine staff who are rude. A 43-year-old female stated:

*“…Majority of malaria vaccine staff are very polite and caring. However there are some staff who are rude. One day my children were sick, I went to Magunga Hospital (Magunga is the name of Korogwe District Hospital) I was so embarrassed by one doctor. I am not sure whether he is the project employee or the government employee but I thank God some malaria vaccine staff assisted me…”* (Participant number A06)

The majority of respondents, especially in the FGDs, expressed that in case of death of any of the study participants the malaria vaccine project does participate in the funeral. Participants commented that the project always provides condolences and transport for caretakers of the study participants in case of any death. A 59-year-old male respondent commented that:

*“…Malaria vaccine project do provide transport and condolences to the caretakers in case of any death of the study participants…”* (Participant number A13)

All participants in both FGDs and IDIs explained that the malaria vaccine project provides free treatment to the community and especially to those trial participants. Free treatment is perceived by respondents as one of the primary benefits established by MVTs. One 48-year-old female commented:

*“…Even if the parents or guardians do not have money they still get quality health service, this project does not segregate between rich people and poor people…”* (Participant number A45)

The majority of the respondents in both FGDs and IDIs explained that the malaria vaccine project has established a referral system for participants who cannot be treated at the project dispensary or at Korogwe District Hospital. This particular theme was discussed by respondents in both FGDs and IDIs. Respondents expressed that once the study participant is seriously sick and he/she cannot be treated at the health facility across the study area, the patient is referred immediately to a referral hospital for further check up and treatment. The community perceived this as a vital secondary health benefit brought by the malaria vaccine project. A 43-year-old female explained:

*“…One day my child had a foreign body ingestion (chicken bone) he couldn’t be treated at Korogwe District Hospital. The project referred my child to KCMC Referral Hospital and he was well treated and is doing very fine now”* (Participant number A06)

### Transport

Overall, the majority of participants in all FGDs and IDIs stated that the availability of transport is one of the major secondary health benefits brought by the project. Most participants explained that project vehicles provide free transport to study participants, as well as non-study participants. As one 59-year-old male stated:

*“…Malaria vaccine project provide free transport to the people…”* (Participant number A26)

A few respondents were quiet on the above topic, maybe because they were not involved directly in the research or because they stayed closer to the project dispensary. Fewer participants, especially in the IDIs, expressed that the project collaborated well with the District Hospital in case of emergencies. A 36-year-old male health worker explained:

*“…For example last year during the national vaccination campaign malaria project has given us the vehicle and driver. They really assisted the Hospital. We are very grateful for the collaboration which exists between the project and the Hospital…”* (Participant number B55)

The majority of respondents, especially in the FGDs, were not conversant with the collaboration between the project and the District Hospital because they were not involved directly in the day-to-day activities of the project as well as the District Hospital.

### Human resources

A few participants, especially in the IDIs, explained that the project has massively increased the number of health professionals across the study area. Respondents claimed that the project employed qualified staff from different parts of the country as well as from outside the country. A 36-year-old male health worker commented:

*“…There are project staff who are working in the paediatric ward and radiology unit; they really assist to bridge the gap of the shortage of human resources which we are facing in this hospital…”* (Participant number B55)

Another 36-year-old male health worker explained:

*“…If I can recall correctly we came here in 2006, in Korogwe District Hospital there was no medical doctor but MVT has been able to employ three qualified medical doctors and 12 nursing officers who are working in the paediatric ward”* (Participant number B54)

A few respondents, especially in the IDIs, expressed that the project had been able to provide employment chances to people in the community across the study area. Respondents explained that the community appreciates the contribution of the project for employing people from the community and they perceive this contribution as one of the major secondary health benefits that has been established by the malaria project. A 36-year-old male health worker claimed:

*“…The presence of malaria vaccine project has assisted the community members to get some auxiliary employment, I have seen some young people employed by the project, although they don’t have good education but these young people could have been bad people in the community but due to the presence of the malaria vaccine project these young people have managed to get employment…”* (Participant number B55)

The majority of respondents, especially in the FGDs, were not conversant with the two subthemes above, perhaps because they were not closely involved in the health systems. Key informants had sufficient information about the topics.

### Infrastructure

A few participants, especially in the IDIs, explained that the project had built nine vaccination centres in the study area and the modern laboratory, which is fully equipped with both modern equipment and qualified staff. Respondents elaborated that the presence of health care facilities across the study area has brought health care services close to the community, which is perceived by the community as one of the most important secondary health benefits established by the malaria vaccine project. A 36-year-old male health worker explained:

*“…The community has been able to get health care services closer to the areas in which they live. In our community it is normal to find the distance between one household and the health facility is too far but the project has managed to build dispensaries closer to the communities which facilitate easier provision of health services…”* (Participant number B55 in the IDI number 06)

The same respondent continued:

*“…Also malaria vaccine project has been able to renovate our dispensaries and they have built some new structures for vaccination of their study subjects. Our dispensaries were in bad condition and old but the project has assisted to renovate. At Korogwe District Hospital the malaria project has built a big modern laboratory…”* (Participant number B55)

The majority of respondents, especially in the FGDs, were not familiar with the above subtheme. They were not sure whether the infrastructure had been built by the government or by the project.

### Misconceptions

A few respondents raised some questions with regard to the trial activities, especially on blood sampling; for example, some respondents claimed that there were reports that the blood collected from participants was sold by the research institute; and others stated that the blood is used for satanic purposes (*Mumiani*- Kiswahili word which means blood-suckers). Those respondents claimed that people with less experience of research, such as the elderly and residents of remote villages, were believed to have spread such rumours.

*“…The amount of blood which is taken is high, so people are scared; they need to be educated. They said the project is selling the blood*…” (Participant number A33)

After further exploration, the majority of the respondents reported that the blood is used for diagnostic purposes especially for malaria disease.

## Discussion

This study found that the RTS,S MVTs had several secondary health benefits as perceived by community. The trials have improved health care services, either directly or indirectly, for the study communities. Respondents explained that since the establishment of the project, the communities involved in the malaria research are pleased with the health care services that are offered by the malaria project. This is not a surprising finding as it is well documented that in a situation of poor service delivery, as in most less-developed countries, medical care in a research setting is described in more favourable terms [2].

Additionally, participants observed that most project staff are polite and caring compared to those working in other health care systems. This observation could be due to the fact that most staff employed by the project are well motivated by continuous training, which they receive on ethical issues and on patient health care. On the other hand, health care workers working in other health care systems, such as in government health facilities and faith-based organizations (FBOs), may be perceived to be rude due to a heavy workload, poor working conditions, or a lack of training in patient health care provider relationships. It is well documented that politeness and caring towards patients by health care providers are very crucial in delivering quality health services [6,11]. These observations underline the importance of training of all health care providers on patient-centred care to improve the quality of health care [12].

This study found that the community perceived the number of deaths of children under five, especially those children participating in the MVTs, had been reduced across the study area. This may be due to the close follow up of young children and prompt attention when need arises by the study staff.

The findings of this study reveal excessive appreciation for the malaria project from the respondents with regard to health care services being offered to the communities by the project. This could be due to lack of trial knowledge among community members because the most commonly provided benefits were medical care (for example free care), and tea or soft drinks while waiting to see a physician. All cash given to participants was reimbursement of transport costs (for example to meet appointments or facilitate use of services when unexpectedly sick), but these payments were often described by research participants as benefits because communities had been receiving poor health care services from other existing health care systems in the country before and even during the implementation of the malaria project. Sometimes it was surprising to learn that the community would like the malaria project to be permanent because of the services offered by the project. This observation should be an alert to other health care systems to improve health care services to poor, rural communities. Similar findings have been reported by Masiye et al., 2008 in Malawi, which reported that a majority of respondents wished to join the project due to the secondary health benefits offered by the project, including soap and transport fare.

Another significant finding in this study is that the trials employed competent health staff and increased the number of health care professionals in the study area. In vaccine trials it is important to employ sufficient staff for the effective implementation of the project. Staff have been employed to work for the malaria vaccine project but they also work for the community surrounding the study area. Availability of health staff employed by the project has been a big boost to the government health facilities, which were overloaded by routine health care activities. This calls for the government to put into place strategies to absorb the additional workforce once the project is over.

The perceived improvement in health care services is similar to the findings from studies in Kenya and Papua New Guinea (6,7) respectively, which reported that the projects were able to improve health care services for trial participants.

The findings of the study also disclosed that the malaria vaccine projects have managed to build new structures and renovate some old buildings across the study area in order to facilitate trial activities.

The presence of health care facilities and other health care investments across the study area has put health care services closer to the community, which is perceived by the community as one of the most important secondary health benefits established by the malaria vaccine project. The government of Tanzania has a policy of building health facilities a maximum 5 km from people, but this is not the case in the Korogwe site because there are villages within the study area which are 15 km from a dispensary.

In addition, the study revealed that there were some misconceptions about MVTs, and particularly rumours regarding blood samples. Some community members were hesitant to participate in the malaria project due to rumours associated with claims that blood was sold by the research institution. This was a misconception and showed a lack of knowledge among community members with regard to research activities. Blood was only used for research purposes and the amount of blood taken considered the health of the participant. However, after further exploration most participants knew that blood samples were used for diagnosis purposes. This particular finding should remind researchers to consider conducting regular sensitization meetings in study communities throughout the study period in order to educate the community about the trial. Community advisory boards (CABs) are effective ways for studies to communicate with the community and will help to clear misconceptions, which, if ignored, will be likely to affect future community participation in clinical trials.

Other studies [2,6,13-15] also reported that participants raised concerns with regard to blood samples. Some community members thought the blood was taken for business and/or satanic purposes. After further exploration, however, the majority of people were aware that the blood was taken for research purposes.

## Conclusion

The study findings showed community appreciation of the secondary health benefits established by the trials in the Korogwe site. The project has led to a massive investment in the health care infrastructure and improvement of health care services across the study area, which was perceived by the community as a major secondary health benefit established by the project. Health care services provided by the project were perceived to be good compared to the government health facilities and FBOs. The project has also been able to increase the workforce in the government health facilities by employing qualified staff working for both trial participants and the community at large. A misconception among respondents, especially with regard to blood samples, was also observed in this study. Sensitization meetings should be regularly conducted by researchers in order to avoid misconceptions in future studies.

## Competing interests

The authors declare that they have no competing interests.

## Authors’ contributions

EL, JL and RM designed the study. EL and RM conducted the study including the analysis of data. EL, JL and RM drafted the manuscript. All Authors reviewed the manuscript and provided critical inputs. All authors read and approved the final manuscript.
